# Upregulation of MIF as a defense mechanism and a biomarker of Alzheimer’s disease

**DOI:** 10.1186/s13195-019-0508-x

**Published:** 2019-06-07

**Authors:** Si Zhang, Jiehao Zhao, Yuhu Zhang, Yun Zhang, Fang Cai, Lijuan Wang, Weihong Song

**Affiliations:** 1 Townsend Family Laboratories, Department of Psychiatry, The University of British Columbia, 2255 Wesbrook Mall, Vancouver, BC V6T 1Z3 Canada; 2 Department of Neurology, Guangdong Neuroscience Institute, Guangdong General Hospital, Guangdong Academy of Medical Sciences, Guangzhou, Guangdong China

**Keywords:** Alzheimer’s disease, Amyloid, MIF, Microglia, Neuronal toxicity, Cognitive impairment

## Abstract

**Background:**

Macrophage migration inhibitory factor (MIF) is a pro-inflammatory cytokine. Chronic inflammation induced by amyloid β proteins (Aβ) is one prominent neuropathological feature in Alzheimer’s disease (AD) brain.

**Methods:**

Elisa, Western blot, and immunohistochemical staining analysis were performed to examine the level of MIF protein in CSF and brain tissues. MTT and LDH assays were used to examine the neurotoxicity, and the Morris Water Maze test was performed to examine the cognitive function in the MIF^+/−^/APP23 transgenic mice.

**Results:**

MIF expression was upregulated in the brain of AD patients and AD model mice. Elevated MIF concentration was detected in the cerebrospinal fluid of AD patients but not in that of the patients suffering from mild cognitive impairment and vascular dementia. Reduced MIF expression impaired learning and memory in the AD model mice. MIF expression largely associates with Aβ deposits and microglia. The binding assay revealed a direct association between MIF and Aβ oligomers. Neurons instead of glial cells were responsible for the secretion of MIF upon stimulation by Aβ oligomers. In addition, overexpression of MIF significantly protected neuronal cells from Aβ-induced cytotoxicity.

**Conclusion:**

Our study suggests that neuronal secretion of MIF may serve as a defense mechanism to compensate for declined cognitive function in AD, and increased MIF level could be a potential AD biomarker.

## Introduction

Alzheimer’s disease (AD) is the most common cause of dementia. It is characterized by the intracellular neurofibrillary tangles, extracellular neuritic plaques, and neuronal loss. Extracellular neuritic plaques are the unique feature distinguishing AD from other forms of dementia and neurodegenerative diseases. Amyloid β protein (Aβ), the central component of neuritic plaques, is generated from the amyloid precursor protein by β– and γ–secretase cleavages [1, 2]. One of the common features of AD neuropathology is the activation of microglia and neuroinflammation. Aβ deposits are particularly potent in the activation of microglia [3]. Despite clear evidence of the recruitment of microglia to the vicinity of plaques, there are ongoing debates on the function of the recruited microglia and how microglia participate in Aβ deposition and clearance during AD pathogenesis. In addition, microglia are the primary immune cells responsible for cytokine production, including pro- and anti-inflammatory cytokines as well as growth factors [4], thus potentially serving as a double-edged sword in response to stimuli depending on the physiopathological status of the brain.

Macrophage migration inhibitory factor (MIF) is a pleiotropic protein that participates in many cellular activities and plays an essential role in regulating the inflammatory response, energy metabolism, and apoptosis. MIF has been shown to be beneficial in promoting survival of cardiomyocytes during cardiac ischemia/reperfusion (I/R) by inhibiting apoptosis, reducing ROS production and regulating glucose metabolism [5–7]. Previously, we have shown upregulation of MIF by hypoxia in the acute phase of stroke [8, 9] and demonstrated the protective role of MIF in suppressing oxidative stress-induced caspase-3 activation [10]. MIF has also been identified as a PARP-1 (Poly (ADP-Ribose) Polymerase 1)-dependent AIF (apoptosis-inducing factor)-associated nuclease (PAAN), and disruption of MIF’s nuclease activity inhibited cell death induced by glutamate excitotoxicity and focal stroke [11]. In AD pathogenesis, neuronal death through apoptotic pathways is observed, and there is evidence suggesting that oxidative stress originating from dysregulation of mitochondria functions serves as a cause of apoptosis [12]. Therefore, MIF may be essential for neuronal survival during AD pathogenesis. In contrast, MIF could also play a deleterious role when overexpressed by immune cells, resulting in excessive inflammation in chronic inflammatory diseases in the systems outside of the CNS [13]. Whether MIF plays a role in initiating and/or maintaining the inflammatory status in the CNS remains unknown. In AD patients, Aβ deposits induce chronic neuroinflammation which features in the activation of resident microglia and infiltration of peripheral macrophages [14]. However, the current understanding of the expression regulation of MIF and its role in AD is limited. This study aimed to investigate how MIF would participate in these seemly paradoxical roles during AD pathogenesis.

## Materials and methods

### Cerebrospinal fluid (CSF) and brain tissues

CSF samples were obtained from patients visiting the Guangzhou General Hospital. The control group included patients who had no history or evidence of cognitive decline. CSFs were taken by lumbar puncture under anesthesia when the subjects had surgery for diseases other than inflammatory diseases of the central nervous system. CSFs from patients with AD, mild cognitive impairment (MCI), and vascular dementia (VD) were collected for neurological diagnosis. Table 1 listed the sample information regarding the sample size, sex, and age in each group. Frozen control and AD human cortices were obtained from the Department of Pathology, Columbia University. These samples were used to examine the expression of MIF by immunoblotting experiments. Table 2 listed the sample information regarding the sex, age, and brain areas used in the study.

**Table 1:** Patient information for the CSF analysis

Subjects	Sample size (female/male)	Age (mean ± SD)
Control	30 (14/16)	61.2 ± 9.6
AD	28 (14/14)	69.4 ± 6.8
MCI	10 (4/6)	68.9 ± 9.6
VD	17 (8/9)	66.3 ± 10.2

**Table 2:** Patient information for the brain tissue analysis

Code	Group	Sex	Age (years)	Area
1751	AD	M	76	Fc
1780	AD	M	72	Fc
4556	AD	F	70	Fc
4693	AD	F	70	Fc
4854	AD	M	54	Fc
794	Control	F	51	Fc
1170	Control	M	58	Fc
1226	Control	M	23	Fc
1441	Control	M	51	Fc
4263	Control	M	61	Fc

*AD* Alzheimer’s disease, *M* male, *F* female, *IB* immunoblotting, *Fc* frontal cortex

### Animals

Animal experiment protocols were approved by The University of British Columbia Animal Care and Use Committee. APP23 transgenic mice carry human APP751 cDNA with the Swedish double mutation at positions 670/671 (KM→NL) under control of the murine Thy-1.2 expression cassette [15, 16]. The PS45 transgenic mice carry human presenilin-1 cDNA with the G384A mutation [17]. *Mif*^*+/−*^ mice on the c57/BL6 background were generated by breeding *Mif*^*+/−*^ mice on BALB/c background (The Jackson Laboratory), with in-house bred c57/BL6 mice. All mice were allowed to access water and food ad libitum. APP23/PS45 double transgenic mice were bred by cross APP23 with PS45 mice. APP23/MIF^+/−^ mice were bred by cross APP23 with *Mif*^+/−^ mice on c57/BL6 background. Positive pups were determined by genotyping [18].

### The Morris water maze test

The Morris water maze test was performed as previously described [19]. Briefly, the test was performed in a 1.5-m diameter pool with a 10-cm diameter platform placed in the southeastern quadrant of the pool. The procedure consisted of 1 day of visible platform tests and 4 days of hidden platform tests, plus a probe trial 24 h after the last hidden platform test. In the visible platform test, mice were tested for five continuous trials with an inter-trial interval of 60 min. Mouse behavior including distance traveled and escape latency was automatically video-recorded by automated video tracking (ANY-maze, Stoelting). The tests were performed on APP23/MIF^+/−^ mice, and APP23 mice, which were negative littler mates of APP23/MIF^+/−^ mice. The tests were performed at the ages between 13 and 14 months.

### Immunohistology

Half brains were fixed in 4% PFA in PBS. Fixed brains were either dehydrated in 30% sucrose solution followed by cryosectioning at 30 μm thickness or prepared for paraffin-embedded sectioning at 5 μm thickness. For immunohistochemistry, the brain slices were incubated with 3% hydrogen peroxide in PBS for 10 min, permeabilized in 0.3% Triton X-100 in PBS (PBS-Tx) for 30 min, and blocked with 5% BSA in PBS for 1 h at 22 °C. Next, the slices were incubated with primary antibodies at 4 °C overnight. After rinsing with PBS-Tx for three times, the slices were applied with secondary antibodies for 1 h at 22 °C, followed by 30-min incubation with avidin-biotin-peroxidase complex (ABC, Vector Laboratories). Color development was achieved by the DAB (Vector Laboratories) method. After rinsing with ddH_2_O, brain sections were subjected to additional hematoxylin staining to visualize nuclei. The sections were observed under traditional microscopy. For Aβ plaque detection, the procedure for secondary antibody incubation was omitted, and the slices were proceeded for ABC incubation and color development. The number of plaques was quantified manually following qualification under × 40 magnification. The area of the plaques was quantified using ImageJ. Images were taken from 10 matching areas (5 slices with 540-μm intervals for each mouse) between APP23 and APP23/MIF^+/−^ transgenic mice. All the images used for plaque quantification were taken at the same time with the same exposure level. For immunofluorescent staining, the brain slices were permeablized in PBS-Tx for 30 min followed by sequential incubation with primary and fluorescent-labeled secondary antibodies as above. After rinsing with PBS, brain sections were coverslipped using the VECTASHILD® mounting medium with DAPI (Vector Laboratories) and observed under fluorescent microscopy. The primary antibodies are rabbit anti-MIF antibody (Torrey Pines Biolabs), mouse anti-GFAP antibody, biotinylated 4G8 antibody, rabbit anti-Iba-1 (DAKO). Secondary antibodies are biotinylated swine anti-rabbit IgG (DAKO) for immunohistochemistry, Alexa 488-labeled goat anti-rabbit IgG (Invitrogen), Alexa 594-labeled goat anti-rabbit IgG (Invitrogen), Alexa 488-labeled goat anti-mouse IgG (Invitrogen), and Alexa 594-labeled goat anti-mouse IgG (Invitrogen) for immunofluorescent staining. Primary and secondary antibodies were diluted in PBS with 1% BSA.

### Immunoblotting

Cortical tissues were homogenized by sonication with 5X (*v*/*w*) RIPA-DOC lysis buffer supplemented with a complete mini protease inhibitor cocktail tablet (Roche Molecular Biochemicals). The samples were then centrifuged at 16,000×*g* at 4 °C for 30 min. The supernatants were removed and added to 2X Novex® tricine SDS sample buffer (Invitrogen) followed by boiling at 100 °C for 2 min. The samples were resolved in 12% tris-tricine gels and transferred to PVDF-FL membranes (Millipore). The membranes were blocked with 5% non-fat milk and incubated with primary antibodies for MIF (Torrey Pines Biolabs) and β-actin (Sigma, AC-15). To detect the proteins, IDye680-labeled goat anti-rabbit and IDye800-labeled goat anti-mouse antibody were used. The blots were scanned using the Odyssey Imager (Licor).

### Cell culture, Aβ oligomer preparation, ELISA, LDH, and MTS assays

The mouse microglia cell line BV-2, mouse macrophage cell line RAW264.7, human neuroblastoma cell line SHSY-5Y, and a stable cell line overexpressing MIF (SYMS) were maintained in Dulbecco’s modified Eagle’s medium supplemented with 10% fetal bovine serum, 1 mmol/L of sodium pyruvate, and 2 mmol/L of l-glutamine (Invitrogen). Cells were seeded onto 96-well plates and cultured at 37 °C in an incubator supplemented with 5% CO2. Aβ oligomers were prepared as previously described with modification [20, 21]. Briefly, synthetic Aβ1-42 was dissolved in 1,1,1,3,3,3-hexafluoro-2-propanol (HFIP, Fluka), vacuum dried, and dissolved in DMSO as a 5 mM stock. Aβ oligomer was prepared by diluting the stock Aβ in sterile PBS to 100 μM and incubated at 4 °C for 12 h. The oligomers were further diluted to 10 μM or 50 μM by culture medium to treat the cells. LPS was used as a positive control for MIF secretion and was used at the concentration of 100 ng/mL. Sixteen hours after Aβ treatment (10 μM), the culture medium was collected and centrifuged at 3000×*g* for 2 min at 4 °C prior to assays. MIF concentrations in culture media were measured by a human (R&D systems) or mouse (Mybiosource) MIF ELISA kit following the manufacturer’s instruction. Culture media were diluted five and two times prior to the assays to measure human and mouse MIF concentration, respectively. To assess cell membrane integrity, LDH assay (Promega) following the manufacturer’s instruction was performed using the same batch of culture medium. SHSY-5Y and SYMS were treated with Aβ oligomers (50 μM). To detect Aβ-induced cytotoxicity, MTS assay was performed following the manufacturer’s protocol (Promega).

### Dot blot assay

To prepare the membrane for the dot blot assay, 2 μL of oligomerized Aβ peptide (100 μM) or purified green fluorescent protein (GFP) protein (approximately 50 μM) were spot on a nitrocellulose membrane and were let dry. The membrane was then blocked in 0.3% BSA in PBS for 1 h at room temperature prior to incubation with mixed proteins of purified hMIF and GFP at the concentration of approximated 5 μM at 4 °C for overnight. The membrane was then washed, and immunoblotting was performed to detect MIF and GFP. The primary antibody to detect MIF was a monoclonal anti-MIF antibody (D-2, Santa-Cruz). The primary antibody to detect GFP was a polyclonal anti-GFP antibody.

## Results

### Upregulation of MIF expression in AD

**Fig. 1:**
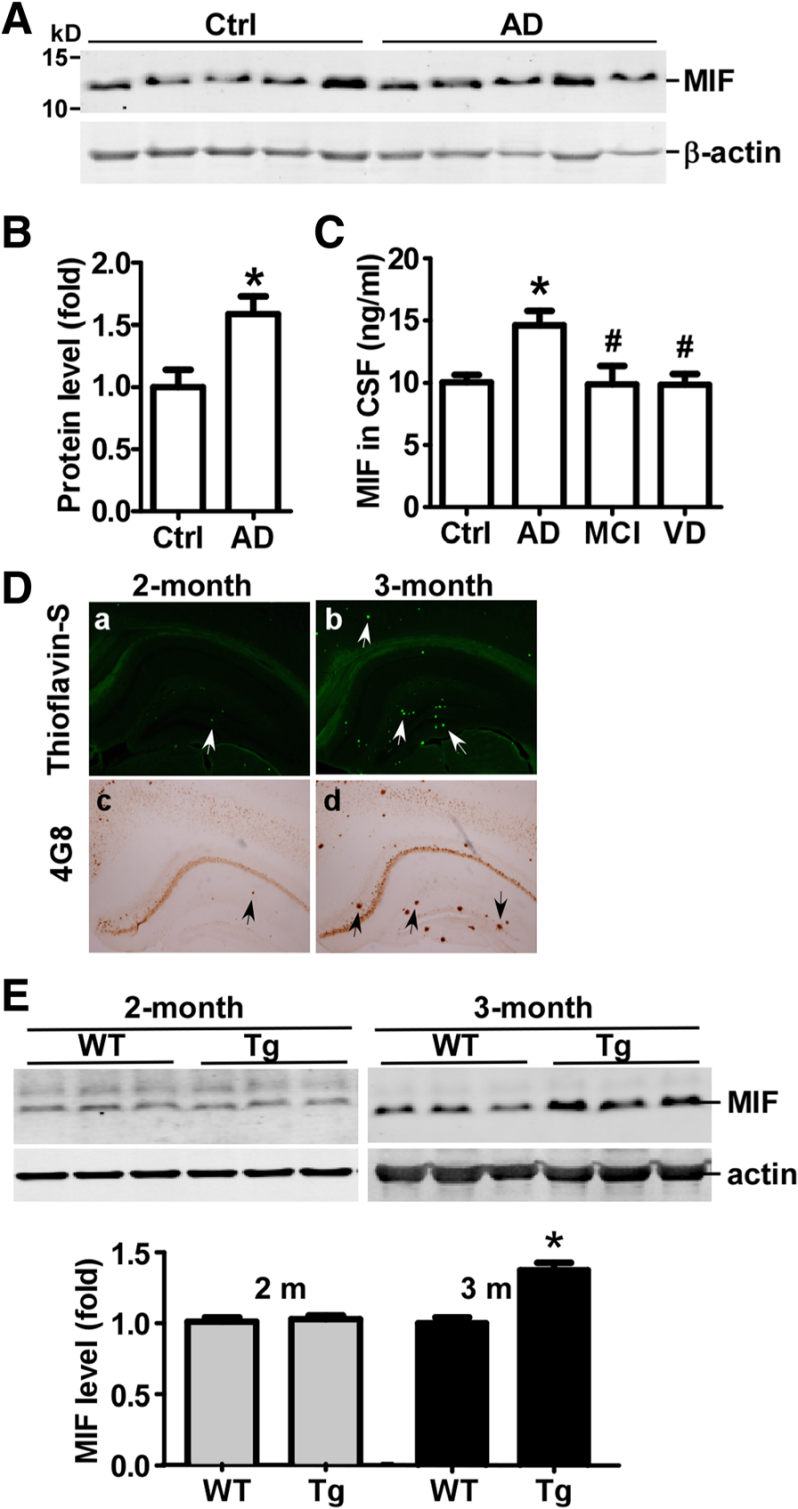
Upregulation of MIF in AD patients and AD model mice. **a** Human brain tissues obtained from Columbia University were lysed in RIPA-DOC buffer, and an equal amount of protein was resolved on a 12% tris-tricine gel. MIF was detected by anti-MIF antibody, and β-actin was detected by the β-actin antibody. **b** Quantification of (A). Values were expressed as mean ± SEM, *n* = 5. *p < 0.05 by Student’s *t* test. **c** Concentrations of MIF in CSF collected from patients with MCI, AD, and VD, and control subjects were measured by ELISA. Values were expressed as mean ± SEM, *n* = 30 for control, 28 for AD, 10 for MCI, and 17 for VD. *p < 0.05 by one-way ANOVA with Newman-Keuls post hoc tests compared to control. #*p* < 0.05 by one-way ANOVA with Newman-Keuls post hoc tests compared to AD. **d** APP23/PS45 double transgenic mice and the wildtype controls were euthanized at the ages of 2 and 3 months. Half of the brain was fixed and sectioned for plaque assessment, and the other half was used for MIF expression evaluation. Thioflavin S (**a**, **b**) and 4G8 (**c**, **d**) were used to stained representative brain sections for plaque detection. Arrows point to neuritic plaques. **e** Brain tissues were lysed in RIPA-DOC buffer, and an equal amount of protein was resolved on a 12% tris-tricine gel. MIF was detected by anti-MIF antibody, and β-actin was detected by β-actin antibody serving as the loading control. The ratio of MIF to β-actin was normalized to wildtype mice. Values were expressed as mean ± SEM, *n* = 4~8 for wildtype mice and 4~10 for APP23/PS45 mice. *p* > 0.05 by Student’s *t* tests. (I) *p < 0.05 by Student’s *t* tests

Postmortem cortical tissues collected from AD patients and controls were assayed by immunoblotting to evaluate the expression of MIF (Fig. 1a). The expression level of MIF was significantly elevated for 1.58 ± 0.14 folds of the controls (*p* < 0.05) (Fig. 1b). To assess the MIF level in AD patients, CSF was collected from control subjects and patients diagnosed with MCI and AD. CSF from patients with VD was also collected. The age and sex distribution of the patients are listed in Table 2. Our results showed that the MIF level in the CSF was significantly upregulated in the patients diagnosed with AD (14.62 ± 1.15 ng/ml) compared to the control subjects (10.07 ± 0.60 ng/ml) (*p* < 0.05) (Fig. 1c). There were no significant differences in the concentration of MIF between the MCI, VD patients (9.89 ± 1.48 and 9.86 ± 0.83 ng/ml, respectively) and the control subjects (*p* > 0.05). However, the MIF level in the MCI and VD patients was significantly lower than that in AD patients (*p* < 0.05) (Fig. 1c). Notably, the CSF concentration of MIF elevated approximately 1.4 folds in AD patients compared to the that in control subjects, which was similar as the fold increase in the brain tissue, indicating the level of MIF in CSF could be an indicator of the level of MIF in the brain tissue. Our results demonstrated that MIF was upregulated in AD, and the elevation of MIF in CSF could be a biomarker of AD.

To determine the change of the MIF expression during AD pathology, we measured the expression of MIF in the brain tissue from APP23/PS45 double transgenic mice at different ages. APP23/PS45 mice have an accelerated AD-like pathological progression [17, 22, 23], and by the age of 3 months, the mice have developed a significant amount of plaques and cognitive impairments in behavioral tests. Brain slices obtained from APP23/PS45 mice were stained with thioflavin S and 4G8 for plaque detection. In 2-month old mice, a few plaques appeared in the cortical and hippocampal regions and were scattered in the cortical layers (Fig. 1d). As expected, the number of plaques significantly increased at 3 months of age in all brain regions as indicated by both thioflavin S and 4G8 staining (Fig. 1d). MIF expression levels in cerebral parenchyma were also measured using the same group of mice. Immunoblotting results showed that MIF protein level in the brain tissue was similar between wildtype and APP23/PS45 mice at the age of 2 months (1.00 ± 0.03 vs 1.02 ± 0.02 folds, *p* > 0.05) (Fig. 1e). However, at 3 months of age, MIF expression in the APP23/PS45 mice increased to 1.37 ± 0.05 fold (*p* < 0.05) of the WT mice at the same age (Fig. 1e). Our results demonstrated that upregulation of MIF in the APP23/PS45 mice occurred at the late stage of the disease when a large amount of amyloid plaques had been formed.

### Increased MIF secretion protects neuronal cells from Aβ-induced neurotoxicity

**Fig. 2:**
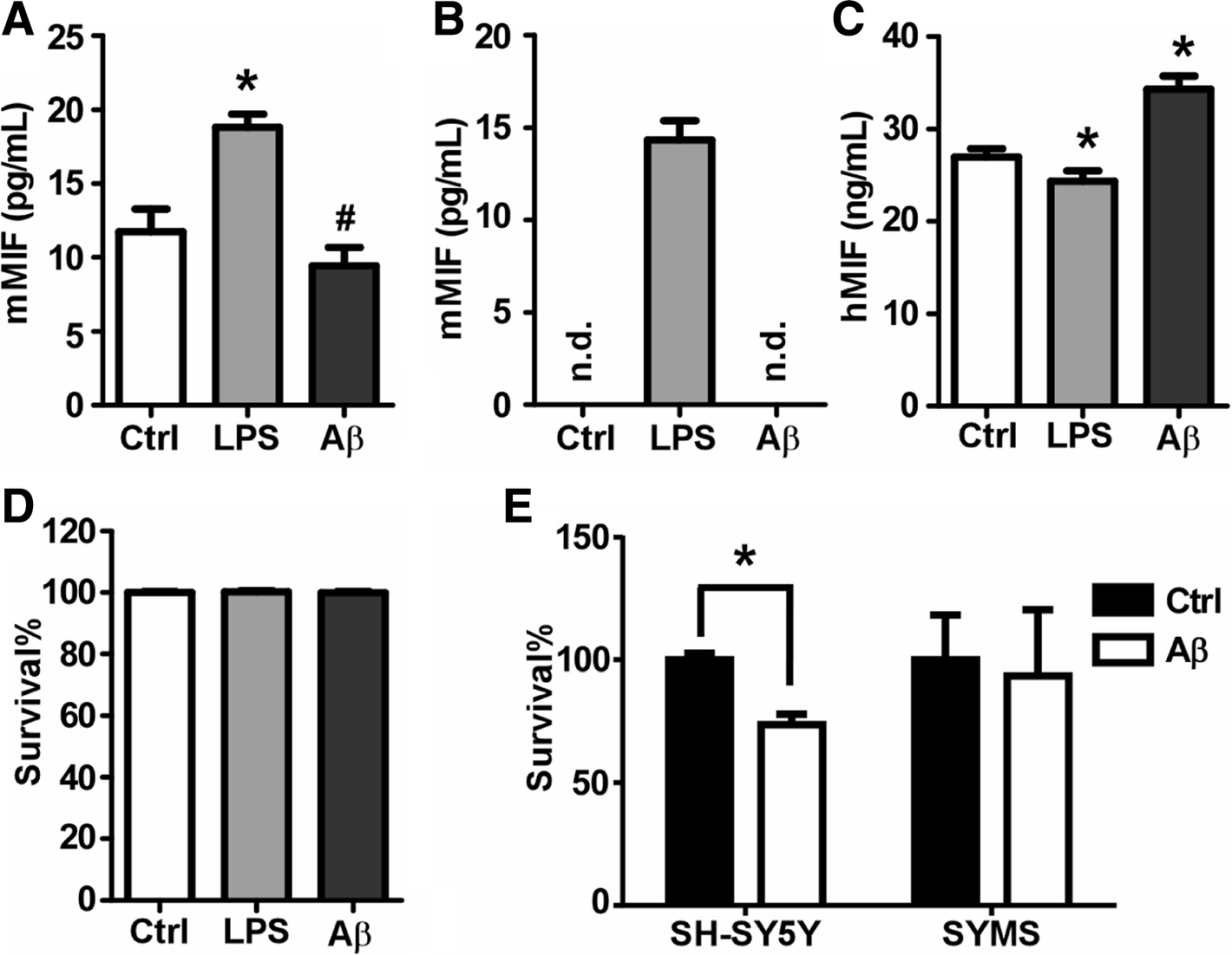
Increased MIF secretion protects neuronal cells from Aβ-induced neurotoxicity. Cells were seeded on seeded onto 96-well plates and cultured 24 h prior to treatment. Aβ treatment was achieved by adding medium diluted Aβ1-42 oligomer stock solution (100 μM in sterile PBS) at the final concentration of 10 μM or 50 μM. LPS at the concentration of 100 ng/mL was used as a positive control for MIF secretion. Sixteen hours after treatment, the culture medium was collected and centrifuged prior to analysis. Media collected from RAW 264.7 (**a**), BV-2 (**b**), and SHSY-5Y (**c**) cell lines were measured for MIF concentrations by ELISA. Values represent mean ± SEM, *n* = 4. *p < 0.05 relative to controls by one-way ANOVA with Newman-Keuls post hoc tests. #*p* < 0.05 relative to LPS by one-way ANOVA with Newman-Keuls post hoc tests. **d** The same batch of media from SHSY-5Y cells were subjected to LDH assay to evaluate the cell membrane integrity. Values represent mean ± SEM, *n* = 4. *p < 0.05 relative to controls by one-way ANOVA with Newman-Keuls post hoc tests. **e** SH-SY5Y and SYMS cell lines were subjected to Aβ oligomer treatment at the final concentration of 50 μM. After 24-h treatment, cell viability was assessed by MTS assays. Values represent mean ± SEM, *n* = 3. *p < 0.05 relative to controls by two-way ANOVA with Bonferroni’s multiple comparison test

MIF is a secretory protein and exerts functions via autocrine/paracrine mechanisms. To examine whether Aβ could trigger MIF secretion, we first tested whether MIF could be secreted after Aβ treatment on mouse microglia and mouse macrophage cell lines, BV-2 and RAW 264.7, respectively. LPS was used as a positive control as it triggers MIF secretion in RAW 264.7 [24, 25]. Baseline expression of MIF was detected in the medium from RAW 264.7 at the concentration of 11.73 ± 1.54 pg/mL (Fig. 2a), while not detectable in that from BV-2 (Fig. 2b). After the LPS treatment, the concentration of MIF significantly increased in the culture medium of both cell lines to 18.83 ± 0.88 ng/mL (*p* < 0.05) (Fig. 2a) for RAW 264.7 and 14.35 ± 0.86 ng/ml for BV-2 (*p* < 0.05) (Fig. 2b). However, Aβ treatment did not significantly alter the concentration of MIF in the medium from RAW 264.7 (9.43 ± 1.24 pg/mL) compared to controls, but it was significantly lower than that from LPS treatment (*p* < 0.01) (Fig. 2a). To our surprise, Aβ treatment did not elevate the concentration of MIF above the detection limit in the culture medium from BV-2 (Fig. 2b). LDH assays were performed to examine the cell membrane integrity that could affect the release of MIF. Our result demonstrated that Aβ treatment did not result in cell membrane leakage in both cell lines.

To examine neuronal secretion of MIF by Aβ, we treated SH-SY5Y cells. The baseline release of MIF to the culture medium from the SH-SY5Y was also observed at the concentration of 26.98 ± 0.91 ng/mL (Fig. 2c). In contrast to the increased MIF secretion in RAW264.7 and BV-2, LPS treatment slightly reduced MIF release in SH-SY5Y to the concentration of 24.36 ± 1.12 ng/mL, compared to controls (*p* < 0.05) (Fig. 2c). The Aβ treatment on SH-SY5Y cells induced a significant increase of MIF concentrations in the culture medium to 32.97 ± 0.79 ng/mL (Fig. 2c), and the increase was not due to the cell membrane leakage as shown by LDH assay (Fig. 2d). Taken together, our result suggested that Aβ-triggered MIF secretion by neurons.

Next, we examined whether elevated MIF secretion could protect neuronal cells from Aβ-induced toxicity. We generated an SH-SY5Y cell line stably overexpressing MIF (SYMS). We treated the SH-SY5Y and SYMS cells with Aβ oligomers (50 μM). After 24 h, the MTS assay was performed to detect cell viability. We found that Aβ treatment reduced the cell survival rate to 73.64% ± 2.49% in SH-SY5Y cells. However, there was no significant difference in cell viability between Aβ-treated SYMS cells and untreated ones, suggesting that elevated MIF secretion protects neurons from Aβ-induced cytotoxicity (Fig. 2e).

### MIF deficiency impairs cognitive functions

**Fig. 3:**
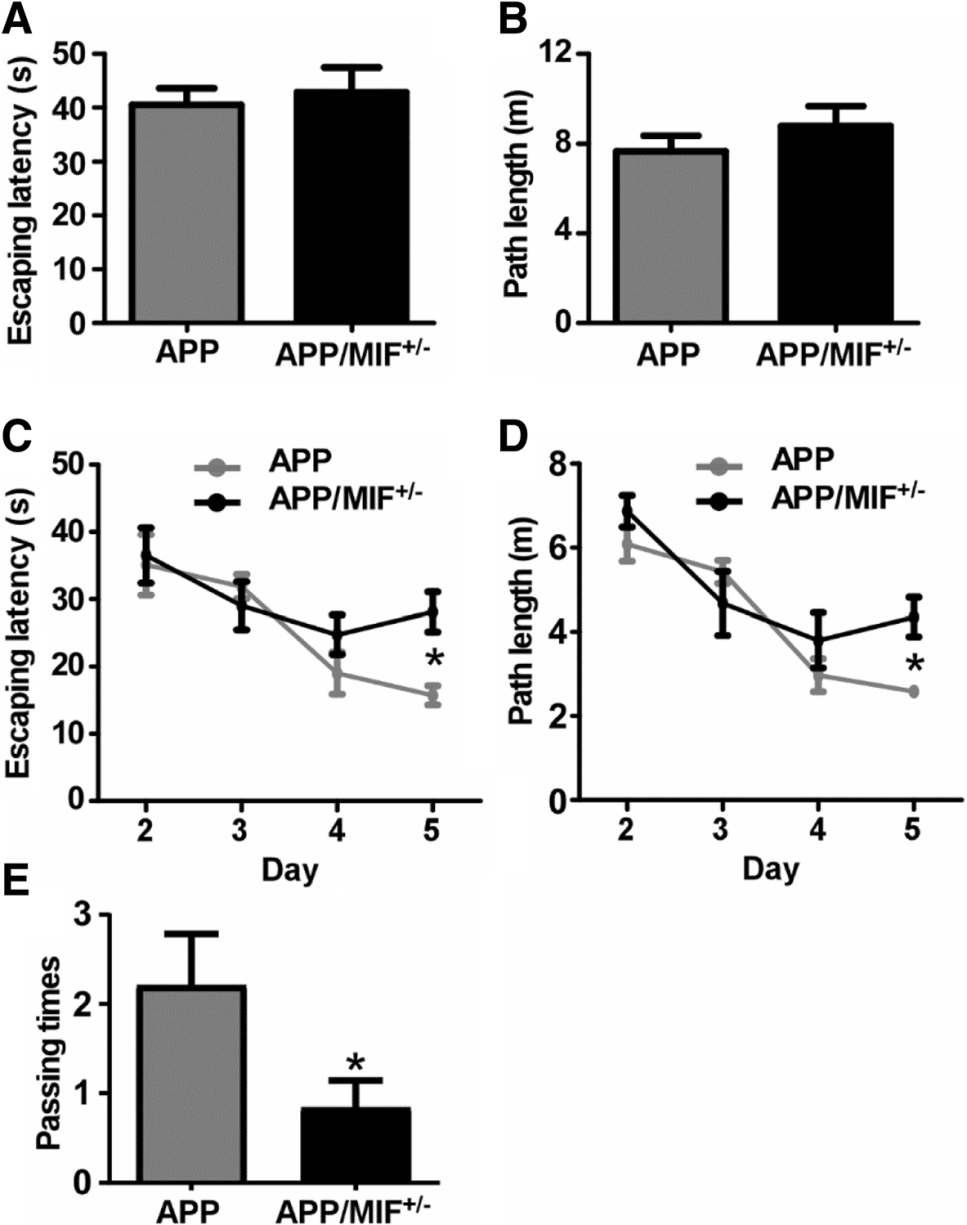
MIF deficiency affects cognitive functions in the AD model mice. APP23/MIF^+/−^ and APP23 mice (APP/MIF^+/−^ and APP, respectively) at the age of 12 to 13 months were subjected to the Morris water maze test. **a** During the first day of visible platform tests, the APP23/MIF^+/−^ and APP23 mice exhibited a similar latency to escape onto the visible platform. *P* > 0.05 by Student’s *t* test. **b** APP23/MIF^+/−^ and APP23 mice had similar swimming distances before escaping onto the visible platform. *P* > 0.05 by Student’s *t* test. **c** In hidden platform tests, APP23/MIF^+/−^ mice showed a longer latency to escape on to the hidden platform on the 5th day. *p < 0.05 by two-way ANOVA with Bonferroni post hoc tests. **d** APP23/MIF^+/−^ mice had a shorter swimming length before escaping onto the hidden platform on the 5th day. *P < 0.05 by two-way ANOVA with Bonferroni post hoc tests. **e** On the last day of the trial, APP23/MIF^+/−^ showed a significantly lower number of passing times through the location of the platform than APP23 mice. *P* < 0.05 by Student’s *t* test

The above results suggested that neurons were responsible for the MIF secretion triggered by the Aβ deposits. To test whether MIF participates in cognitive performance during AD pathogenesis, APP23/MIF^+/−^ and APP23 mice were subjected to memory function assessment by the Morris water maze at the age between 12 and 13 months. In the visible platform tests, APP23/MIF^+/−^ and APP23 mice had similar escape latencies (40.5 ± 3.0 s and 42.8 ± 4.6 s, *p* > 0.05) (Fig. 3a) and path length (7.7 ± 0.7 m and 8.7 ± 0.7 m, *p* > 0.05) (Fig. 3b), indicating that hemizygous knockout of MIF did not affect mouse mobility or vision. In the hidden platform test, APP23/MIF^+/−^ mice showed significant memory impairment on the fifth day of the test. The escape latency on the last day of the hidden platform test was significantly longer than in APP23 mice (28.1 ± 2.9 vs 15.7 ± 1.4 s, *p* < 0.05) (Fig. 3c). The APP23/MIF^+/−^ mice also swam significantly longer distances to reach the platform as compared to APP23 mice (4.3 ± 0.5 vs 2.6 ± 0.1 m, *p* < 0.05) on the fifth day of hidden platform test (Fig. 3d). In the probe trial on the last day of testing, the platform was removed. APP23/MIF^+/−^ mice showed a significantly lower number of times passing through the position of the hidden platform than APP23 mice (*P* < 0.05) (2.2 ± 0.6 vs 0.8 ± 0.3, *p* < 0.05) (Fig. 3e). These results indicated that MIF deficiency affected spatial learning during the hidden platform training.

### MIF expression is associated with amyloid plaques

**Fig. 4:**
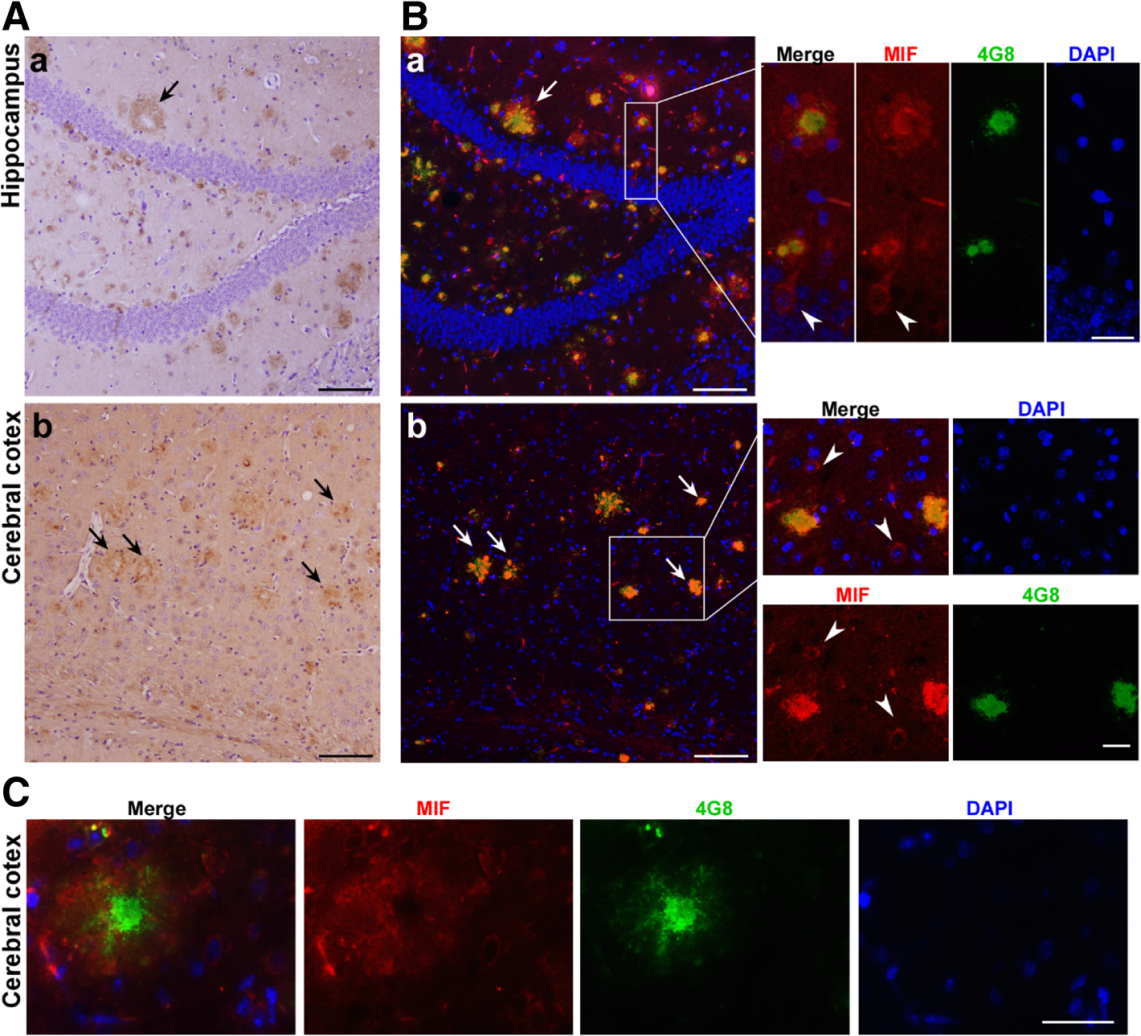
Colocalization of MIF and amyloid plaques in APP/PS mice. **A**, **B** Fixed brains were prepared for paraffin-embedded sectioning at 5 μm thickness. MIF was detected by anti-MIF antibody and visualized by ABC and DBA methods. Nuclei were stained by hematoxylin. The sections were observed under traditional microscopy. Arrows point to plaques associated MIF expression, which resembles the expression pattern of Aβ plaques. Scale bar, 100 μm. **C** Fixed brains were dehydrated in 30% sucrose solution and embedded in O.C.T. for cryosectioning at 30 μm thickness. MIF was detected by polyclonal MIF antibody, and Alexa 488-labeled secondary antibody, and Aβ plaques were detected by monoclonal 4G8 antibody and Alexa 594-labeled secondary antibody. Nuclei were stained with DAPI, and brain sections were observed under fluorescent microscopy. Arrowspoint to colocalization of MIF and plaques. Arrow heads point to MIF expression cells. Scale bar, 100 μm in B, and 25 μm in inserts and C

Since our results suggested that sufficient MIF is necessary for normal cognitive performance, it seems controversial that AD patients with increased MIF levels still demonstrated memory loss. In order to address this discrepancy, we thoroughly analyzed the expression patterns of MIF in the brain. Immunohistology was performed on brain sections from APP23/PS45 mice using a specific anti-MIF antibody. The pattern of MIF staining demonstrated as spotted clusters in both the hippocampal region and cerebral cortex (Fig. 4A), which remarkably resembled that of 4G8 stained Aβ plaques. Next, we performed the double staining of MIF and 4G8 on an adjacent brain section (5 μm) to the one used for the DAB staining. The fluorescent signals for MIF as pointed by white arrows (Fig. 4B) were confirmed by the DAB staining of MIF as pointed by black arrows (Fig. 4A). The double staining of MIF and 4G8 revealed the colocalization of MIF and Aβ plaques (Fig. 4B). Interestingly, two patterns of association between MIF and Aβ plaques were observed. Besides the colocalization as shown in Fig. 4B, b, some MIF staining exhibited a pattern that embraced the dense core of the Aβ plaques in hippocampal (Fig. 4B, a, insert) as well as in cortical (Fig. 4C) regions. There were a few cells stained positive for MIF (arrowheads, Fig. 4B inserts), and we did further analysis to identify the cell type.

**Fig. 5:**
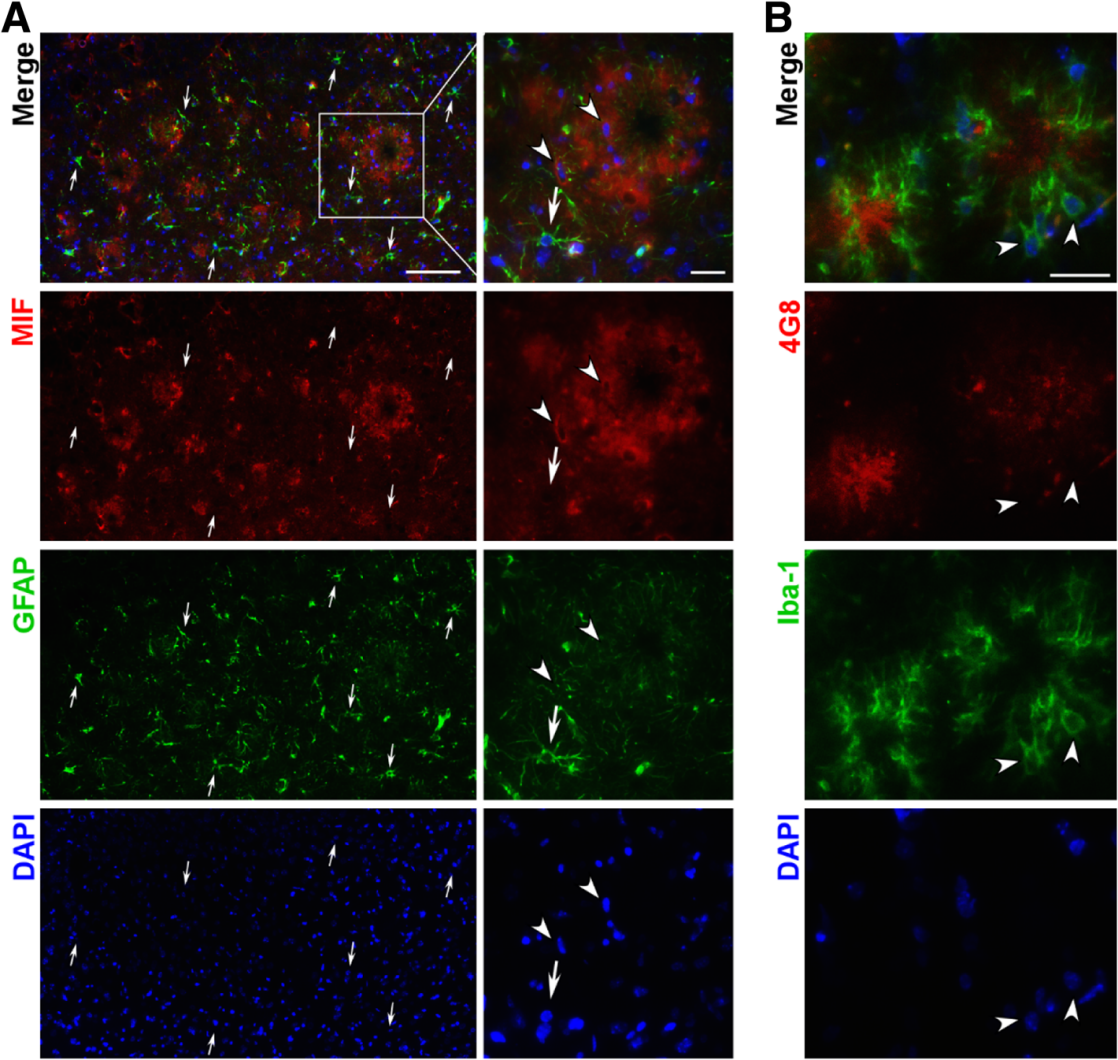
MIF expression overlaps but is not restricted in the vicinity microglia and astrocytes. Fixed brains were dehydrated in 30% sucrose solution and embedded in O.C.T. for cryosectioning at 20 μm thickness. **a** MIF was detected by polyclonal MIF antibody and Alexa 488-labeled secondary antibody, and active astrocytes were detected by monoclonal GFAP antibody and Alexa 594-labeled secondary antibody. Nuclei were stained with DAPI, and brain sections were observed under fluorescent microscopy. Arrows point to GFAP-positive cells. Arrowheads point to possible MIF expressing cells. Scale bar, 200 μm and 50 μm in inserts. **b** Neuritic plaques were detected by monoclonal 4G8 antibody and Alexa 488-labeled secondary antibody, and microglia were detected by polyclonal Iba-1 antibody and Alexa 594-labeled secondary antibody. Nuclei were stained with DAPI, and brain sections were observed under fluorescent microscopy. Arrowheads point to possible MIF expressing cells. Scale bar, 50 μm

Since MIF expression was tightly associated with amyloid plaques, we examined whether glial cells were responsible for the plaque-associated MIF expression. Double staining of MIF and GFAP was performed on brain sections from APP23/PS45 mice at the age of 7 months (Fig. 5a). A similar MIF expression pattern was observed, confirming the characteristics of MIF expression in the AD brains—enriched in the vicinity of amyloid plaques. Active astrocytes stained with GFAP were prevalent in the entire brain section without particular enrichment. At the site of MIF enrichment, MIF expression overlapped with GFAP staining, although the highest expression of GFAP was neither in close vicinity of nor colocalized with MIF (arrows, Fig. 5a). Due to a lack of available antibodies, double staining for colocalization study was not performed for MIF and microglia. Instead, amyloid plaques were stained with 4G8 to locate possible expression of MIF, and Iba-1 was used to detect microglia (Fig. 5b). Our result demonstrated that Iba-1 positive microglia were in closer vicinity to the amyloid plaques than astrocytes, suggesting highly possible overlapping of microglia and MIF expression. In addition, the morphology of MIF expression cells (arrowheads, Fig. 5a) was very similar to Iba-1 stained microglia (arrowheads, Fig. 5b).

### MIF interacts with Aβ oligomers

**Fig. 6:**
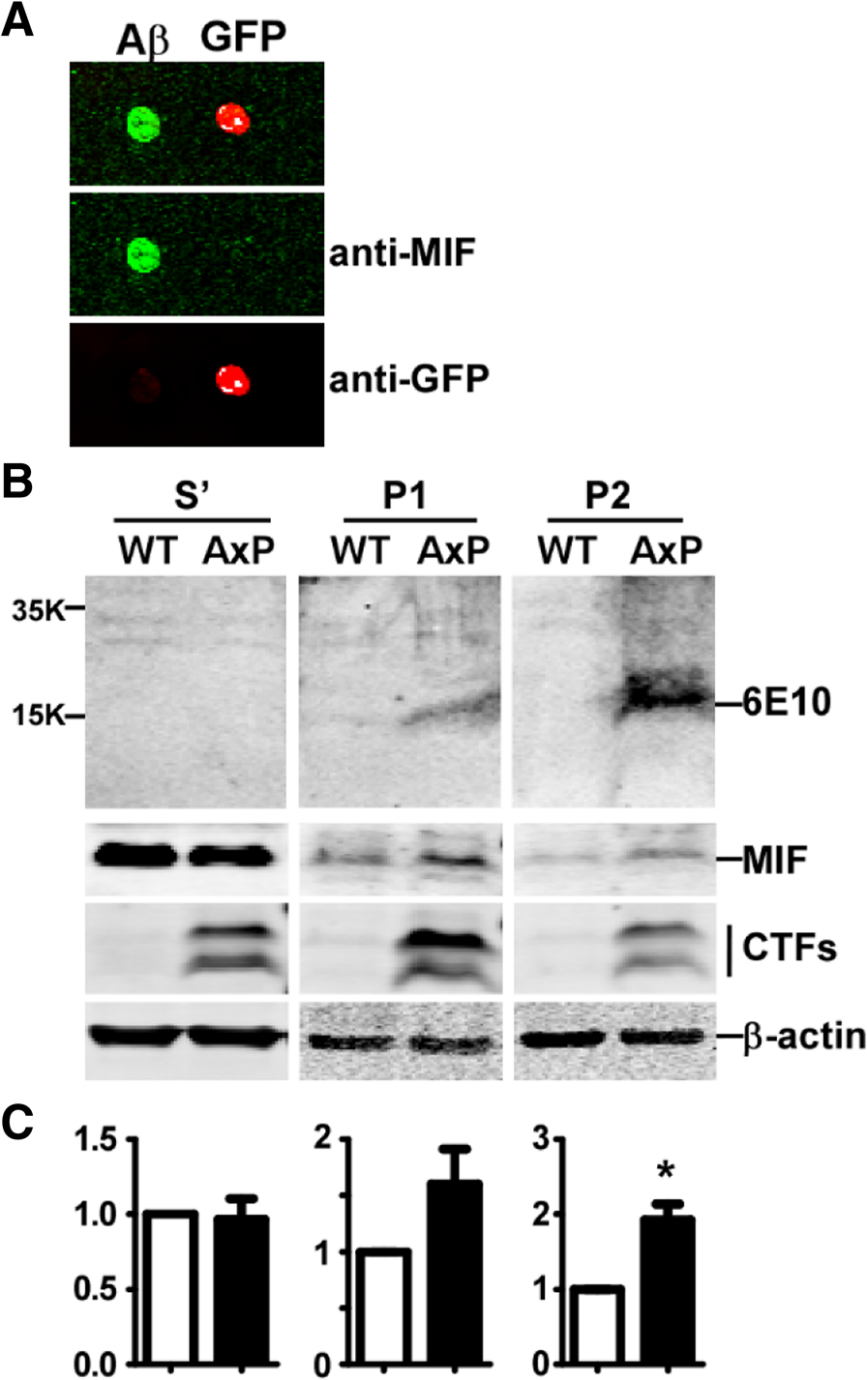
MIF interacts with Aβ oligomers. **a** 0.2 nmol of Aβ oligomers and 0.1 nmol of purified GFP protein were spot on a nitrocellulose membrane, and the membrane was incubation with mixed proteins of recombinant hMIF and purified GFP at the concentration of approximated 5 μM at 4 °C for overnight. The membrane was then subjected to immunoblotting to detect MIF and GFP by a monoclonal anti-MIF antibody and a polyclonal anti-GFP antibody, respectively. The red channel detects IR-dye-labeled goat anti-rabbit antibody, and the green channel detects IR-dye-labeled goat anti-mouse antibody. **b** Brains from 4-month-old APP23/PS45 and wildtype (as controls) mice were homogenized in 5x PBS with 0.5% Triton-100 (*v*/*w*) and centrifuge at 16,000×*g* at 4 °C for 15 min. The supernatants were collected and subjected to ultracentrifugation at 100,000×*g* for 1 h at 4 °C, and the second supernatants were collected and labeled as S′. Homogenates from each layer were further dissolved in RIPA-DOC buffer followed by brief sonication. The same amount of protein was loaded on 16% and 12% Tris-tricine SDS PAGE gels for Aβ and MIF separation, respectively. Aβ was detected by a 6E10 antibody, MIF was detected by anti-MIF antibody, CTFs were detected by a C20 antibody to confirm the expression of the transgene, and β-actin was detected by β-actin antibody serving as the loading control. S′, supernatant after ultracentrifugation; P1, pellet 1; P2 pellet 2. **c** Quantification of the level of MIF protein from **b**. Values represent mean ± SEM, *n* = 3. *P < 0.05 by Student’s *t* test

Our results strongly suggested a possible association of MIF with amyloid plaques in the extracellular space. However, one could not rule out the possibility that the presence of MIF was merely due to constant secretion by the vicinity cells. In order to examine whether MIF is associated with plaques in the extracellular matrix, we first examined whether MIF could interact with Aβ using a dot blot assay. Oligomerized Aβ and GFP were spot on a membrane and incubated with the protein mixture of MIF and GFP. GFP spotted on the membrane served as the control for non-specific binding by MIF, and GFP in the protein mixture served as the control for non-specific binding by Aβ. The result demonstrated that MIF interacted with oligomerized Aβ, but neither did MIF nor Aβ interact with GFP, indicating a specific binding between oligomerized Aβ and MIF (Fig. 6a). We then studied whether MIF and Aβ were associated in the AD brain by exploring whether MIF and Aβ present at the same density fraction. Brain tissues were obtained from APP23/PS45 mice and homogenized in PBS with 0.5% Triton X-100. The homogenates were fractionated under 16,000×*g* for 15 min at 4 °C. The homogenates were clearly separated into four layers, namely supernatant (S), pellet layer 1 (P1), pellet layer 2 (P2), and pellet layer 3 (P3). The supernatants were collected and subjected to ultracentrifugation at 100,000×*g* for 1 h at 4 °C, and the second supernatants were collected and labeled as S′. The layer P3 mainly consisted of non-uniform tissue debris was discarded. Subsequently, samples collected from S′, P1, and P2 fractions were subjected to immunoblots to locate the presence of Aβ enriched fractions. To study the potential association between plaques and MIF, immunoblots were performed to detect the presence of Aβ and the level of MIF in each collected fraction. Our results demonstrated that Aβ was not detectable in the S′ fraction, but was detected in P1, P2, and P3 fractions (Fig. 6b). Concentrations of MIF were similar between controls and the APP23/PS45 mice in the supernatant, indicating AD pathology did not increase the level of free MIF in the soluble fraction (0.96 ± 0.14 fold of the control, *P* > 0.05) (Fig. 6b, c). Interestingly, soluble MIF tended to be enriched in the P1 fraction (1.60 ± 0.31 fold of the control, *P* > 0.05) and was significantly enriched in the P2 fraction (1.92 ± 0.21 fold of the control, *P* < 0.05), where Aβ aggregates presented (Fig. 6b, c). Taken together, these results are supportive that MIF could bind with Aβ plaques in AD brains.

## Discussion

Overproduction of Aβ leads to chronic inflammation that is characterized by activation of microglia cells and increased production of inflammatory mediators in the brain, such as pro-inflammatory cytokines, chemokines, macrophage inflammatory proteins, and prostaglandins [14]. MIF as a pro-inflammatory cytokine has been shown to be upregulated systematically in circulation and locally at lesion sites in many chronic inflammatory diseases and plays central roles in disease progression. Previous studies have shown that MIF was upregulated in plasma and CSF from AD patients [26–29]. In the present study, we demonstrated that MIF level was increased in the postmortem cortical tissues and CSF of AD patients and the late stage of the AD transgenic mouse brains with a substantial amount of Aβ deposits. Notably, the MIF level was not altered in either CSF from patients with MCI or brain tissues from young AD transgenic mouse brains, suggesting that MIF probably was not immediately upregulated at the early stage of AD pathology. This result is in line with a previous study showing that among a series of pro-inflammatory cytokines including MIF, only the production of IL-1β was induced in 14-month old APP23 single transgenic mice, which developed a few Aβ deposits [30]. Interestingly, MIF level was not elevated in patients with VD, indicating the elevation of MIF in the CSF possibly is specific to patients with dementia due to AD. This was indicative that MIF release could be a defense mechanism by neurons, and that was how AD is distinguished from other types of dementia, such as VD.

In addition to temporal expression pattern, our study further characterized the spatial expression pattern of MIF. In our study, we clearly demonstrated that MIF expression immediately surrounds Aβ plaques. Furthermore, we showed that MIF directly binds with Aβ. Protein associated with Aβ may affect fibril formation and deposition. For example, cystatin C has been reported to associate with Aβ42 to inhibit the formation of amyloid fibrils [31]. Cystatin C and MIF share a similar tertiary structure with 4-strand β-sheet, and both undergo self-aggregation amorphologically or to filaments at a physiological condition and are amyloidogenic at lower pH in vitro [32, 33]. Because MIF binds to Aβ directly and colocalizes with Aβ plaques, whether MIF could affect the amyloidogenesis of Aβ warrants further studies.

It has been suggested that MIF facilitates defending mechanisms under stressful conditions by promoting cell survival in the systems outside the CNS. During heart ischemia, MIF acts in an autocrine fashion and signals through the CD74/44 receptor complex to promote cell survival by temporarily maintaining energy homeostasis; knockout of MIF thus results in the larger ischemic damage [7]. In the CNS, as we demonstrated, maintaining MIF expression is important in defending cerebral ischemia by reducing oxidative stress-induced caspase-3 activation in neurons during the acute phase [10]. Maintaining MIF expression could also be important for neurons to defend Aβ-induced toxicity. However, there is limited research carried out to investigate the effect of MIF in Aβ-induced toxicity in neurons, except for one study demonstrating a protective effect of a MIF inhibitor on Aβ-induced toxicity in SH-SY5Y cells [26]. In contrast to their results, we identified that Aβ stimulated the secretion of MIF from SH-SY5Y cells and increased secretion of MIF significantly reduced neuronal cell death induced by Aβ. It has been shown that inhibition of p53 attenuates Aβ-induced neuronal apoptosis [34], and MIF can directly inhibit p53 activation [35]. Thus, secretion of MIF precedes cell damage, and MIF is released under the autocrine fashion, in turn, activates cell survival signals.

It should be noted that MIF acts in an autocrine fashion and interacts with its cell surface receptors to transduce its signals [36, 37]; therefore, secretion of MIF to the extracellular space is necessary for MIF to exert its cellular functions. It has been suggested that MIF is constitutively expressed and is perhaps secreted constantly by neurons in the brain [38, 39]. However, at the late stage of AD, a large portion of the extracellular MIF is sequestered by Aβ plaques, as we demonstrated, and perhaps has no functions anymore, which suggests that increased MIF secretion is essential for maintaining cognitive function during AD development. This also explains why MIF upregulation is observed at the late stage of AD with significantly increased Aβ deposition.

We found that neurons specifically secreted MIF following Aβ stimulation. However, we did not rule out the possibility that upregulation of MIF is also contributed by microglia (local or infiltrated) at the late stage of AD, at which pro-inflammatory cytokines are predominantly produced [14], and they are known to trigger secretion of MIF [25]. Indeed, recent studies demonstrated a mixture of hyperactivated microglia at the late stage of AD pathology [40] and the upregulation of MIF in hyperactivated microglia [41]. We speculated that at the late stage of AD pathology, overproduced MIF could still be beneficial in promoting neuronal survival if it can be received by neurons; on the other hand, however, the pool of MIF produced by immune cells may locally promote their own survival and proliferation, which in turn produce and release more MIF, leading to a vicious circle as seen in chronic inflammatory diseases in the peripheral.

Although it is debatable whether Aβ deposits serve as a cause of cognitive decline during AD, inhibition of Aβ production and plaque formation have been shown to successfully mitigate cognitive deficits in AD model mice [17, 18, 42, 43]. Since MIF insufficiency had an impact on cognitive performance [44], we hypothesized that Aβ-triggered MIF secretion could serve as a compensatory mechanism to improve cognitive performance during AD. In contrast, inhibition of MIF has been suggested for treating peripheral inflammatory diseases [13, 26], indicating the possibility that the potential pro-survival and pro-inflammatory functions of MIF counteract each other during AD, and inhibition of one would tip a balance to the other. It is possible that at the early stage with little Aβ plaques formation, MIF secretion triggered by Aβ oligomer is pro-survival for neurons to maintain cognitive function; while at the late stage with abundant Aβ plaques, the compensatory function may fail to demonstrate effects due to the direct binding between MIF and Aβ, leaving behind the pro-inflammatory effects. Therefore, it will be of importance to dissect the MIF signal complex so that the deleterious effects could be inhibited without affecting the beneficial ones.

## Conclusions

In summary, we found that the upregulation of MIF expression specifically occurs in patients with AD rather than other types of dementia and identified that the level of MIF in CSF could serve as a biomarker of AD with global inflammation. In addition, hemizygous knockout of MIF exacerbated memory task performance of APP23 AD model mice. More importantly, we for the first time revealed the direct interaction between MIF and Aβ. Taken together, our data provided first-hand evidence of MIF expression profile during AD and its effect on AD development. Our study suggested that proper regulation of MIF expression, secretion, and function is essential for the successful treatment of AD.

## Data Availability

The authors agree the availability upon request.

## Abbreviations

AD: Alzheimer’s disease
APP: Amyloid β precursor proteins
Aβ: Amyloid β protein
MIF: Macrophage migration inhibitory factor
